# Streptococcal endocarditis: a meta-analysis of species dependant risk

**DOI:** 10.1016/j.eclinm.2025.103425

**Published:** 2025-08-25

**Authors:** Gavin Deas, Todd C. Lee, Julia Colston, Mahableshwar Albur, Julia Vasant, Angela H. Nobbs, Philip Williams, Fergus Hamilton

**Affiliations:** a University Hospitals Bristol & Weston NHS Trust, Marlborough Street, Bristol, BS2 8HW, UK; b Clinical Practice Assessment Unit, Department of Medicine, McGill University, Montreal, H4A 3S1, Canada; c North Bristol NHS Trust, Southmead Hospital, Southmead Road, Bristol, Avon, BS10 5NB, UK; d Royal United Hospital Bath NHS Foundation Trust, Combe Park, Bath, BA1 3NG, UK; e Oral Microbiology, Bristol Dental School Research Laboratories, University of Bristol, Bristol, BS1 3NY, UK; f MRC Integrative Epidemiology Unit, University of Bristol, Bristol, BS6 5ES, UK

**Keywords:** Endocarditis, Infection, Streptococcal species, Bacteraemia

## Abstract

**Background:**

Streptococcal infective endocarditis (IE) is a devastating disease. In international guidance, the risk of IE from streptococci is considered the same regardless of species (excluding *Streptococcus pyogenes* and *Streptococcus pneumoniae*). However, the idea of homogenous risk has been recently questioned. We aimed to evaluate the risk of IE across streptococcal species through meta-analysis of other published works and our own local data.

**Methods:**

We first conducted a scoping review for publications that reported cases of streptococcal bacteraemia differentiated by species and estimated the risk of IE between 1994 and October 2024. Then we supplemented this data with our own laboratory data from four large hospitals. We meta-analysed the risk of IE. Two sensitivity analyses were performed to deal with one manuscript which excluded cultures considered as contaminants: first, by excluding that publication, and second by adjusting for blood culture contamination using our local estimated contamination rates.

**Findings:**

Four studies met inclusion criteria comprising a total of 14,183 isolates with 1028 endocarditis cases (7.25% absolute risk of IE). The highest risk species were: *Streptococcus mutans*: 47% (95% CI 38–56%), *Streptococcus cristatus*: 41% (95% CI 21–62%), *Streptococcus gordonii*: 37% (95% CI 30–44%), *Streptococcus sanguinis* 33% (95% CI 28–39%), and *Streptococcus gallolyticus*: 31% (95% CI 27–36%). Combined, these species accounted for only 8.4% of bacteraemias but 38.6% of IE. The most common IE pathogen overall was *Streptococcus mitis/oralis* (23.6% of IE, 8% of bacteraemias) but these infections themselves only carried an IE risk of 12% (95% CI 11–13%). There was strong evidence of heterogeneity detected in *S. mitis/oralis* (I^2^ 87%; Cochran’s Q: 30 p: <0.001) and *S. gallolyticus* (I^2^ 90%; Q: 29 p: <0.001).

**Interpretation:**

The ‘small five’ streptococci: *S. mutans, S. cristatus, S. gordonii, S. gallolyticus,* and *S. sanguinis* account for only 8% of all streptococcal bloodstream infections but nearly 40% of all streptococcal IE with a risk of IE in individual infections as high as ∼50%. The risk of *S. mitis/oralis* appears heterogeneous, may depend on species or subspecies, and requires further study.

**Funding:**

FH was funded by the NIHR Clinical Lectureship Scheme. TL has salary support from Fonds de Recherche Québec - Santé. PW is funded by a 10.13039/501100000265Medical Research Council grant MR/T005408/1.

**Research in context**

**Evidence before this study**

Endocarditis diagnosis relies on the Duke criteria (1994, modified 2023), which includes major microbiological criteria for streptococci (except *pneumoniae* and *pyogenes*). Recent clinical tools and research by Chamat-Hedemund (Circulation 2020) revealed varying endocarditis rates among different streptococcal species. However, these findings may not apply universally due to differences in diagnostic practices and populations across countries.

To address this, we analysed our data alongside McGill University Healthcare's bacteraemia prevalence data, then searched for additional datasets using PubMed (1994–2024) with MeSH terms for endocarditis and streptococcal infections, applying relevant study design filters. Publications were screened for those recording streptococcal bacteraemia and endocarditis rates by species.

**Added value of this study**

Our study shows that the risk of endocarditis is related to streptococcal species, and there is little heterogeneity across the different studies included, suggesting this is biological reality not artefact of study design. Our total sample size now allows for precise estimates for even rare species. We identified two species that did have statistical heterogeneity: *S. gallolyticus* and *S. mitis/oralis* with the latter being identified as organism of interest in further research.

**Implications of all the available evidence**

Our findings challenge the Duke criteria's classification of most streptococcal species as “typical pathogens” for endocarditis, as the evidence shows up to ten-fold variation in endocarditis risk between different species. Patients should be stratified to echocardiography and further investigation based on the species of streptococci.

## Introduction

Streptococcal bacteraemia is common and forms a large proportion of work for clinical infection specialists and microbiology labs.^1^ The major challenge–particularly with the ‘oral’ streptococci, is identifying the small proportion of patients who have deep infection, particularly infective endocarditis (IE).^2^ Investigation for IE often requires transthoracic and/or transoesophageal echocardiography amongst other investigations, therefore determining the risk of IE in any patient with streptococcal bacteraemia is a significant task.^3^ Currently, the updated 2023 ISCVID Duke criteria for endocarditis specifies that all streptococci (excluding *Streptococcus pneumoniae* and *Streptococcus pyogenes*) be considered typical causative pathogens of IE.^4^

Why some species of streptococci seem to cause endocarditis more than others given their genetic homology is an active area of research but likely relates to specific abilities to adhere to and survive on valvular tissue.^5^^,^^6^ Virulence factors that associate oral or viridans group streptococci with systemic disease point towards extracellular matrix proteins that bind to glucans^7^ and interactions with the host immune response to generate a favourable microenvironment.^8^ These phenomena are not known to be linked to predominant genotypes or lineages amongst those diagnosed with streptococcal IE.^9^ A seminal paper by Chamat-Hedemand et al. suggested that not all streptococci are equal with respect to the risk of IE. Their national data registry cross-linked endocarditis diagnosis, blood culture data, and demographic data to reveal the proportion of endocarditis per streptococcal species at an unprecedented resolution.

Nonetheless, it is possible that the absolute risks of IE in one setting do not transfer into another due to differing patient populations, different degrees of diagnostic stewardship and culture processing approaches to both species identification, or methods of IE diagnosis. Consequently, we aim to better quantify the risk of IE in all patients with streptococcal bacteraemia by pairing a scoping review of the literature with a meta-analysis across the various methodologies. We also include local data from our four hospital network.

## Methods

### Local analysis

All blood culture results positive for the genus *Streptococcus* were extracted from our laboratory system in Bristol, UK, from the 1st January 2017 to 30th June 2024. This laboratory serves 2 large academic teaching hospitals with just over a thousand beds each, and two district general hospitals. Together they make up 2840 inpatient beds of the West of England Pathology network.^10^ Streptococci that could not be identified to species level were excluded, as were duplicate samples: same pathogen in the same patient within 30 days. Available data included: species, location of sampling, and source of infection (as determined by a microbiologist during clinical care). Further details on the blood culture approach are available elsewhere.^1^ Identification of streptococci was made primarily using Matrix Assisted Laser Desorption/Ionisation–Time of Flight (MALDI-TOF, Bruker) and confirmed with subsequent VITEK-2 (bioMérieux) during antimicrobial susceptibility testing.

### Search strategy

Three reviewers (GD, TL and FH) searched for research articles in the database MEDLINE with the MeSH terms: (“Endocarditis”[Mesh]) AND (Streptococcal infections[MeSH Major Topic]) AND (“Humans”[MeSH]).

We searched MEDLINE from 1994 (when the first Duke criteria was established) until Oct 2024.

We (GD and FH) used the following criteria for publications by analysing abstracts or full manuscripts: Inclusion criteria: 1) Reporting streptococcal species prevalence amongst streptococcal bacteraemia, 2) Reporting endocarditis prevalence amongst streptococcal bacteraemia by species 3) Published as Clinical trial, Comparative study, Meta-analysis, Observational study, Research support or Validation study.

Exclusion criteria: 1) Not written in English, 2) Species data unavailable. We performed a scoping review aiming to capture the largest cohorts, but did not perform a formal systematic review. We did not assess risk of bias as part of this work, as there are some unmeasured confounders within the diagnosis of bacteraemia, like vaccination rates or compliance with fluid resistant surgical mask wearing, and within the diagnosis of endocarditis, endocarditis, that are were not reported.

### Statistical analysis

#### Local

We calculated the risk of IE for each streptococcal species by dividing the number of IE cases by the total number of cases of bacteraemia for that species. Due to the trend towards identifying bacteraemia by species, we opted to not use phylogenetic groups such as viridans. The proportion that each species contributed to all cases of streptococcal bacteraemia was calculated by dividing the number of the isolates of each species by the total number of isolates; data for this came from unpublished data from Montreal, our data from Bristol and that published in Chamat-Hedemand (2020).^11^ We only included studies that reported all streptococci to determine the proportion of bacteraemias. As *Streptococcus cristatus* and *Streptococcus sinensis* are closely related, and there were only a few cases for each, we treated them as part of the same group.

#### Meta-analysis

For our main analysis, we performed a fixed-effects inverse variance weighted (IV) meta-analysis of the risk of IE using restricted maximal likelihood estimation using the *meta* package in the statistical software R (ver 4.2.2).^12^ We also report the random effects estimate and heterogeneity statistics.

Our search identified four previous studies. The data from Bristol was added to this to form the meta-analysis. All reported data on the absolute risk of IE as a function of positive blood cultures; however, one study pre-excluded the cultures which they determined to represent contaminants.^13^ This is an issue, as the decision to treat a streptococcal isolate as a contaminant is linked with the decision to investigate it as a potential IE case. By excluding contaminants, the risk of IE per bacteraemia is artificially inflated as the lowest-risk cases are excluded. This was clearly visible as the rate of IE was >2 times higher than all other cohorts. To account for this issue, we planned two sensitivity analyses. Firstly, we compared the results that reported their contamination rates, excluding Fourre et al. Secondly, we used our local data to estimate the contamination rate and used this data to “correct” the denominators in Fourre et al. (Supplementary material 1).

#### Ethics

This was a retrospective audit, with culture information extracted from the laboratory system and identifiable information removed at source, and local data collection was registered as such. Source of infection data was coded and recorded within this as part of routine clinical practice. No patient identifiable data was extracted. As such, ethical approval was not required. This meta-analysis was not registered.

### Role of funding source

Individuals received salary support as outlined in the Acknowledgements section, these funding sources did not have input on the design, administration, data collection, analysis, interpretation presentation or writing of this manuscript.

## Results

### Study screening

**Fig. 1:**
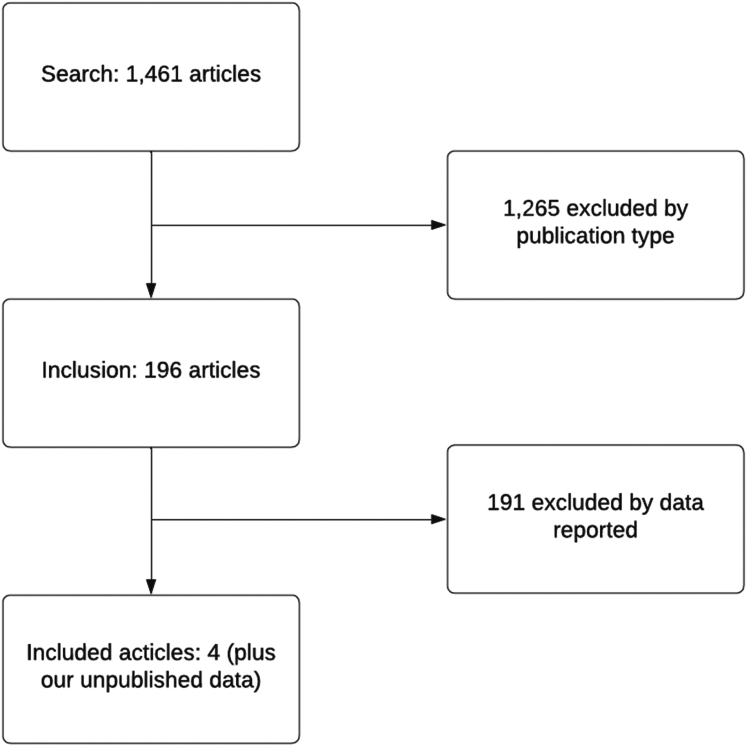
PRISMA flow diagram for article and dataset inclusion.

Three hundred and twenty-eight publications were screened. Four met the inclusion criteria (Table 1; Fig. 1). Chamat-Hedemand (2020)^11^ described Danish national registry data; Seo (2023)^14^ described a South Korean tertiary referral centre data; Sunnerhagen (2018)^15^ described the development of the HANDOC score; and Fourre (2024)^13^ described the validation with the HANDOC score (a clinical decision tool for non-beta haemolytic bacteraemias) with the 2023 Modified Duke criteria for endocarditis. Deas (2024) is this publication. Isolates from McGill totalled 922 and contributed to the species prevalence of bacteraemia; data regarding endocarditis in this cohort was not available.

**Table 1:** Summary of the included studies.

Study	Demographic	Total	IE proportion	ID method	Inclusion criteria	Exclusion criteria
Chamat-Hedemand (2020)	Danish nationwide registries	6506	7.1%	MALDI-TOF	Streptococcal bacteraemia	Paediatric Unregistered Spp not known
Seo (2023)	Tertiary hospital, South Korea	2779	6.4%	VITEK and then MALDI-TOF	Streptococcal bacteraemia	Spp not known
Sunnerhagen (2018)	Tertiary hospital, Sweden	738	7.2%	MALDI-TOF	Non-beta haemolytic streptococcal bacteraemia	Neutropenic Paediatric No records
Fourre (2024)	National registry, Switzerland	894	20.9%^a^	MALDI-TOF	Streptococcal bacteraemia	Contaminants Consent declined
Deas (2024)	Four hospital networks, England	2344	7.7%	MALDI-TOF	Streptococcal bacteraemia	Spp not known

a After adjusting the denominators for contamination, the estimated rate of IE was 9.5%.

### *Streptococcus mitis*/oralis is the most common cause of endocarditis but 5 other species have a higher probability of causing endocarditis

**Fig. 2:**
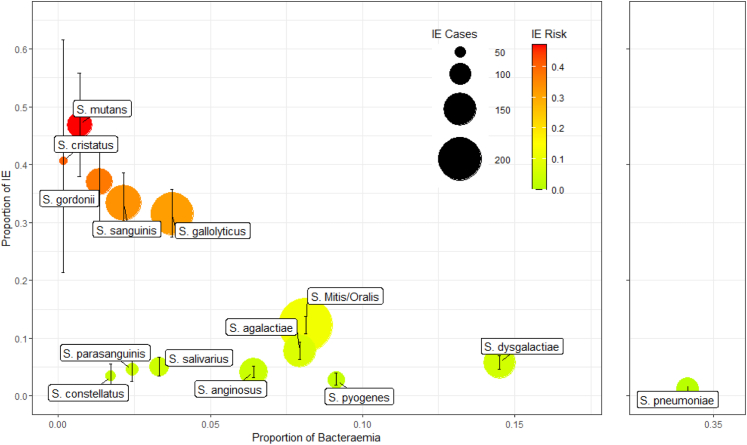
Infective endocarditis prevalence of streptococcal species vs. proportion of streptococcal species bacteraemia. Coloured by increasing endocarditis risk, green (low < 10%) to red (high > 35%). Circles are proportionate to the total number of endocarditis cases. The vertical lines represent the 95% confidence intervals around the proportion of bacteraemias with endocarditis.

A total of 14,183 isolates with 1028 endocarditis cases were included (absolute rate of IE 7.2%, Fig. 2). The most common pathogens causing endocarditis were *S. mitis/oralis* (243), *Streptococcus gallolyticus* (190), *Streptococcus sanguinis* (110), *Streptococcus agalactiae* (92), and *Streptococcus dysgalactiae* (86).

Bacteraemia with five particular species of streptococci had a proportion of endocarditis of greater than 20% of cases, and formed a cluster of high risk streptococci for IE. In descending order these were: *Streptococcus mutans*: risk of IE 47% (95% CI 38–56%), *S. cristatus*: 41% (95% CI 21–62%), *Streptococcus gordonii*: 37% (95% CI 30–44%), *S. sanguinis*: 33% (95% CI 28–39%), and *S. gallolyticus*: 31% (95% CI 27–36%) (Fig. 2). The pyogenic and anginosus group streptococci all presented a low probability of endocarditis in the individual patient, whilst the mitis group includes both the most likely (*S. cristatus*) and one of the least likely (*S. pneumoniae*). It is notable that Seo et al. and Fourre et al. had lower rates of *S. pneumoniae* than the data from Canada, Bristol, or that reported in Chamat-Hedemand (2020), but the proportion of invasive streptococcal disease does not influence the rate IE amongst those species.

### Species specific endocarditis risk is a biological property, rather than diagnostic or patient population

**Fig. 3:**
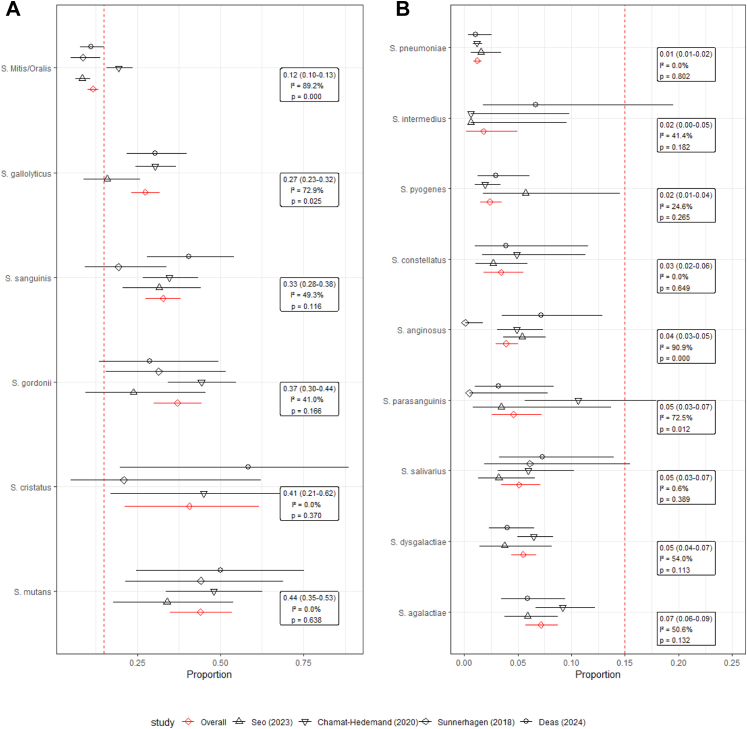
**A and B.** Meta-analysis of each streptococcal species with overall fixed effect, 95% confidence interval, and I ˆ 2 heterogeneity statistics–without adjustment.

**Fig. 4:**
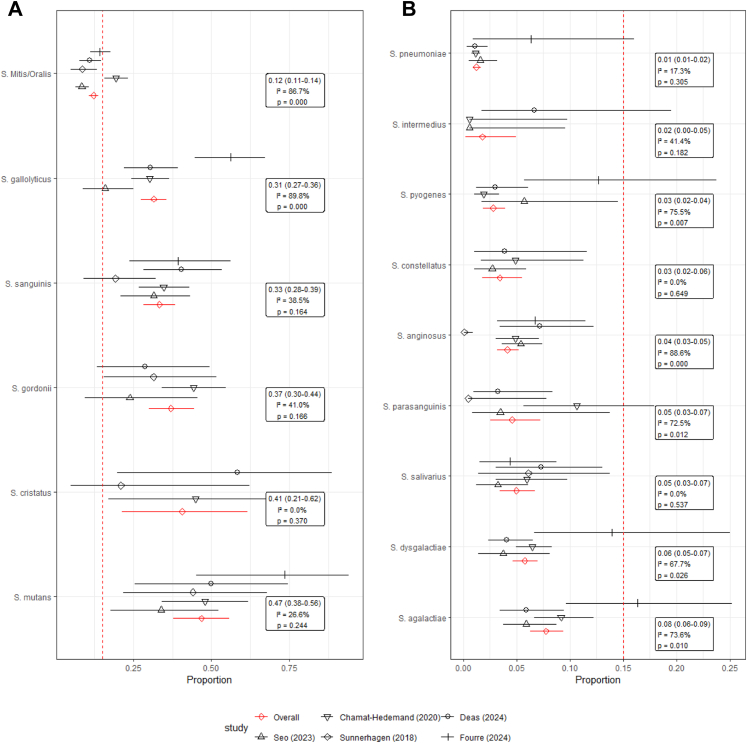
**A and B.** Meta-analysis of each streptococcal species with overall fixed effect, 95% confidence interval, and I ˆ 2 heterogeneity statistics, with Fourre 2024 being adjusted by our local contamination rate.

As shown in Fig. 3, Fig. 4, *S. mitis/oralis* and *S. gallolyticus* have substantial heterogeneity. The correction for contamination also suggested that the beta-haemolytic streptococci were almost never dismissed as contaminants, but the streptococci with the highest risk for endocarditis were often declared contaminants as opposed to other deep-seated infection [Supplementary material 1]. This highlights the paradoxical relationship between highly virulent pathogens like *S. pneumoniae* and *S. pyogenes*, which are classical causes of sepsis but not deep-seated infections like endocarditis.

## Discussion

Our main finding is that there is a group of 5 species of streptococci that accounts for only 8% of streptococcal bacteraemias but 40% of cases of streptococcal IE. Each of these microorganisms, when isolated from the blood, is associated with a high risk of endocarditis for the patient: *S. mutans*: 47% (95% CI 38–56%), *S. cristatus*: 41% (95% CI 21–62%), *S. gordonii*: 37% (95% CI 30–44%), *S. sanguinis*: 33% (95% CI 28–39%), and *S. gallolyticus*: 31% (95% CI 27–36%). In fact, the risk for each of these microorganisms is higher than that for patients with *Staphylococcus aureus* (16%)^16^ or *Enterococcus faecalis* (14%)^17^ bacteraemia. With the potential exception of *S. mitis/oralis*, the other streptococci clearly do not meet that threshold.

The risk seems to defy current phylogenetic grouping and requires further research in terms of their virulence factors and immuno-pathogenesis. One potential reason for this is that the high risk IE bacteria have evolved to primarily bind components within the acquired salivary pellicle under shear forces (unlike *S. pyogenes* and *S. pneumoniae*).^18^ It then seems that the adhesins that contribute to this colonisation confer the capacity to engage with cardiac endothelium and platelets when these microbes enter the bloodstream.^19^ It is also clear that different species can express specific surface determinants that are associated with additional properties related to IE progression; for example, the SRRP adhesin family seems to play a major role for many streptococci. Other specific adhesion factors include: Cnm for *S. mutans*, AsaA for *Streptococcus oralis* and PblA/B for *S. mitis.*^19^ At present it is not clear whether strain specific factors play a role; for example, *S. mutans* serotypes *e* and *f* are strongly linked to endocarditis.^20^^,^^21^ It is also possible that host factors influence the rate of endocarditis, whether that be damaged host tissue, prosthetic material or poor bacteraemia clearance from immunosenescence. Interestingly, the largest species level risk of endocarditis does not match the largest burden of work for cardiologists and infection specialists. *S. mitis/oralis*, *S. dysgalactiae* and *S. agalactiae* all form a large number of IE cases at the population level but pose low to medium risk of endocarditis at the level of individual bacteraemia.

We identified evidence of heterogeneity of risk in *S. mitis/oralis* and *S. gallolyticus*. This may be due to a lack of nuanced classification within these groups. For instance, different subspecies of *S. gallolyticus* may have a different risk of endocarditis and there are likely different proportions of these subspecies within each study.^22^ The same may be true of organisms which classify as *S. mitis/oralis*. Both are notoriously difficult to identify to species level and the majority of the studies utilised MALDI-TOF, which cannot distinguish *S. mitis* from *S. oralis.*^23^ Seo 2023 does suggest a difference in endocarditis risk between the two, with *S oralis* accounting for a third of bacteraemias within this group, but having greater risk of endocarditis. Future work is needed to understand how more granular speciation of these organisms would be of value to more accurately quantify the endocarditis risk, and recently there has been a call to re-evaluate the boundaries of this group.^24^

In our sensitivity analysis we performed two meta-analyses. The first was on all the studies that reported streptococcal bacteraemia that included contaminants. The second was with Fourre 2024 having been adjusted by our contamination rate. We did not detect a difference in heterogeneity because of this, and the overall estimates remained the same.

In summary, only certain streptococcal species should be considered “typical” pathogens within the 2023 ISCVID Duke criteria. This will have important clinical practice implications in terms of management and particularly seeking echocardiography. Further work should be performed to understand the benefit of differentiating *S. mitis/oralis* and within *S. gallolyticus* subspecies, and quantifying the risk, as well as improvising the laboratory methodology to identify them, including specific virulence factors.

Five streptococcal species pose a significant risk in an individual of endocarditis: *S. mutans, S. gordonii, S. cristatus, S. sanguinis* and *S. gallolyticus*; other species could be considered less typical unless clinical factors suggest endovascular infection is likely. The greatest burden of endocarditis comes from *S. mitis/oralis*, which poses only a moderate risk to the individual and the biological heterogeneity within this group is worthy of further investigation.

## Contributors

Data curation GD, TL, FH. Data access and verification GD, TL, FH. Formal analysis GD, TL, FH. Methodology TL, FH. Project administration GD. Supervision FH. Validation TL, FH. Visualisation GD. Writing—original draft GD. Writing—review & editing GD, TL, JC, MA, JV, AN, PW, FH.

## Data sharing statement

Data can be made available with by contacting the lead author at gavin.deas@nbt.nhs.uk. This will be in the form of site, species, endocarditis cases, non-endocarditis cases and years of data collection. Study protocol for analysis and the code in R can be made available by contacting the lead author. This data sharing can be available from publication onwards without restriction.

## Declaration of interests

MA—Merk, Pfizer–consulting fees paid to self. Pfizer–payment or honoraria for lectures, presentations, speakers bureaus, manuscript writing or educational events.

TL—Grants or contracts from any entity (if not indicated in item #1 above) fees paid to Canadian Institutes Health Research—Operating funds and Fonds de Recherche Sante—Quebec—Research salary support.

JC—none, JV—none, PBW—none, FH—none, AN—none, GD—none.
