# Supplementary material for: Streptococcal endocarditis: a meta-analysis of species dependant risk
Source: eClinicalMedicine. 2025 Aug 25;87:103425. doi: 10.1016/j.eclinm.2025.103425 (PMC12496199; doi:10.1016/j.eclinm.2025.103425)
Supplement: Supplementary material 1 [file mmc1.docx]

Supplementary Material 1

|  |  | |  | |  | |  | |  |
| --- | --- | --- | --- | --- | --- | --- | --- | --- | --- |
| Species | | Endocarditis | | Total | | Not IE | | Contaminants | Adjustment factor |
| *S. dysgalactiae* | 15 | | 382 | | 367 | | 0 | | 0 |
| *S. pyogenes* | 6 | | 217 | | 211 | | 0 | | 0 |
| *S. agalactiae* | 15 | | 261 | | 246 | | 0 | | 0 |
| *S. cristatus* | 3 | | 5 | | 2 | | 0 | | 0 |
| *S. pneumoniae* | 5 | | 413 | | 408 | | 2 | | 0.005 |
| *S. gallolyticus* | 33 | | 106 | | 73 | | 3 | | 0.03 |
| *S. constellatus* | 2 | | 63 | | 61 | | 3 | | 0.05 |
| *S. anginosus* | 9 | | 131 | | 122 | | 7 | | 0.05 |
| *S. mutans* | 7 | | 14 | | 7 | | 4 | | 0.29 |
| *S. sanguinis* | 23 | | 57 | | 34 | | 20 | | 0.35 |
| *S. gordonii* | 7 | | 25 | | 18 | | 9 | | 0.36 |
| *S. mitis/oralis* | 34 | | 312 | | 278 | | 139 | | 0.45 |
| *S. salivarius* | 7 | | 102 | | 95 | | 68 | | 0.67 |
| *S. parasanguinis* | 4 | | 107 | | 103 | | 77 | | 0.72 |
